# Identifying the morphologic basis for radiomic features in distinguishing different Gleason grades of prostate cancer on MRI: Preliminary findings

**DOI:** 10.1371/journal.pone.0200730

**Published:** 2018-08-31

**Authors:** Gregory Penzias, Asha Singanamalli, Robin Elliott, Jay Gollamudi, Natalie Shih, Michael Feldman, Phillip D. Stricker, Warick Delprado, Sarita Tiwari, Maret Böhm, Anne-Maree Haynes, Lee Ponsky, Pingfu Fu, Pallavi Tiwari, Satish Viswanath, Anant Madabhushi

**Affiliations:** 1 Department of Biomedical Engineering, Case Western Reserve University, Cleveland, OH, United States of America; 2 University Hospitals, Cleveland, OH, United States of America; 3 Department of Pathology, University of Pennsylvania, Philadelphia, PA, United States of America; 4 St. Vincent’s Prostate Cancer Clinic, Darlinghurst, NSW, Australia; 5 Douglass Hanly Moir Pathology, Macquarie Park, NSW, Australia; 6 Garvan Institute of Medical Research/The Kinghorn Cancer Centre, Darlinghurst, NSW, Australia; 7 Department of Population and Quantitative Health Sciences, Case Western Reserve University, Cleveland, OH, United States of America; University of South Alabama Mitchell Cancer Institute, UNITED STATES

## Abstract

Translation of radiomics into the clinic may require a more comprehensive understanding of the underlying morphologic tissue characteristics they reflect. In the context of prostate cancer (PCa), some studies have correlated gross histological measurements of gland lumen, epithelium, and nuclei with disease appearance on MRI. Quantitative histomorphometry (QH), like radiomics for radiologic images, is the computer based extraction of features for describing tumor morphology on digitized tissue images. In this work, we attempt to establish the histomorphometric basis for radiomic features for prostate cancer by (1) identifying the radiomic features from T2w MRI most discriminating of low vs. intermediate/high Gleason score, (2) identifying QH features correlated with the most discriminating radiomic features previously identified, and (3) evaluating the discriminative ability of QH features found to be correlated with spatially co-localized radiomic features. On a cohort of 36 patients (23 for training, 13 for validation), Gabor texture features were identified as being most predictive of Gleason grade on MRI (AUC of 0.69) and gland lumen shape features were identified as the most predictive QH features (AUC = 0.75). Our results suggest that the PCa grade discriminability of Gabor features is a consequence of variations in gland shape and morphology at the tissue level.

## Introduction

Radiomics involves extracting quantitative features from medical images for disease characterization. [1–3] Specifically radiomic features attempt to capture sub-visual image attributes of the disease that may not be visually discernible. Radiomic approaches have recently demonstrated promise for assessing disease risk *in vivo* in a number of different disease domains including prostate [4–7], breast [8–10], lung [11–14], brain [15, 16], colorectal [17, 18], renal cell carcinoma [19], and head and neck cancer [20, 21]. Different types of radiomic features have been presented in order to capture a variety of different types of information ranging from first and second order image derivatives, to first and second order statistics, steerable gradients, and shape. Haralick features for instance attempt to capture textural appearance based on statistical relationships between gray levels of neighboring pixels [22]; Gabor [23], Sobel, Kirsch, Gauss, and gradient features capture edge orientation information; Hess features [24] capture second order derivatives.

In the context of prostate cancer, radiomic approaches have been presented in order to identify disease presence on magnetic resonance imaging (MRI) and also to non-invasively grade and stratify disease risk. [4–7, 25–28] Since the current clinical gold standard for PCa diagnosis relies on biopsies, which are highly invasive and prone to inaccuracies due to spatial sampling errors, a radiomic approach could potentially provide a safer and more accurate alternative for *in vivo* assessment of PCa risk.

Viswanath et. al. [25] found that Gabor and Haralick features extracted from 3 Tesla (T) endorectal, *in vivo* T2-weighted (T2w) MRI were able to detect prostate cancer (PCa) with area under the curve (AUC) of the receiver operating characteristic (ROC) curve of 0.86 within the central gland and 0.73 within the peripheral zone. Ginsberg et. al. [26] also found Gabor and Haralick features to be useful for detecting PCa on T2w MRI. Litjens et. al. [27] found that Hess, Gabor, and Gauss features extracted from T2w MRI were able to discriminate different benign tumor confounders from PCa with an AUC of 0.7. Fehr et. al. [5] found that a combination of T2w and ADC MRI Haralick texture features were able to distinguish low from intermediate and high Gleason scores with 92% accuracy on a cohort of 147 patients. Additionally, Tiwari et. al. [28] found that a non-linear embedding based fusion approach for combining magnetic resonance spectroscopy (MRS) and directional (Sobel, Kirsch, and gradient) and Haralick texture features of T2w MRI was able to distinguish low from high Gleason grades (a well established surrogate for PCa risk) with an AUC of 0.89 on N = 29 patients.

Successful translation of these radiomic markers into the clinic, however, may require a more comprehensive understanding of the underlying morphologic and histomorphometric underpinning of their ability to dicriminate low- from high-Gleason-score PCa. Unfortunately, and perhaps surprisingly, relatively little work has been presented towards understanding the cellular and morphologic basis of these radiomic features. Ganeshan et. al. [29] assessed correlations between contrast-enhanced and unenhanced computed tomography (CT)-derived radiomic texture and histopathologic markers of tumor hypoxia and angiogenesis in non-small cell lung cancer, and found that radiomic features capturing heterogeneity (such as standard deviation) on contrast-enhanced CT were associated with these histologic markers. The authors note that an important component of intratumoral heterogeneity is blood vessel irregularity, which can result in a heterogeneous vascular supply that can cause areas of hypoxia. The authors suggest that since the radiomic features extracted are all measures of image heterogeneity, the radiomic features might be reflecting hypoxia and angiogenesis, which are histopathologic consequences of intratumoral vascular heterogeneity. Several other studies in other disease domains have identified correlations between similar radiomic features and histopathologic measures of vascularity and hypoxia. [30–32]

Apart from exploring the correlation of imaging features with immunohistochemistry (IHC) attributes of vascularity and hypoxia, some researchers have been looking at the association of pathologist assessment of histologic primitives (e.g. stroma, cellularity, etc.) of PCa with imaging measurements. Langer et. al. [33], in looking at *ex vivo* whole mount histology (WMH) images from radical prostatectomy specimens with pre-operative *in vivo* MRI, found that ADC and T2w MRI were associated with gross measurements of nuclear and lumen space areas; however, none of the parameters or features they evaluated were discriminating of Gleason scores within their cohort. Chatterjee et. al. [34] found that gland component volumes of epithelium, stroma, and lumen space, computed on sub-images of H&E-stained prostate quadrant sections, were more strongly associated with Gleason pattern and ADC than the conventionally cited cellularity metrics of nuclear count and nuclear area. Kobus et. al. [35] found that *in vivo* 1.5-T ADC was correlated with lumen space area and inversely associated with nuclear area on WMH, and lumen space area was correlated with Gleason pattern. The associations these studies identified suggest strong correlations between cellular level tumor features and radiographic measurements. However none of these studies have specifically looked at or considered radiomic measurements from MRI scans.

Similar to radiomic features on radiographic scans, quantitative histomorphometry (QH) features capture morphologic attributes of disease patterns from digitized tissue slide images that may not be visible to a pathologist. [36] Indeed, there have been a number of recent approaches involving QH features for characterizing aggressiveness of PCa on digitized tissue slides. [37–41] For example, Lee et. al. [42] showed that QH features of gland lumen orientations were more disordered in more versus less aggressive prostate cancer, and could more accurately predict biochemical recurrence in PCa than post-operative nomograms. Ali et. al. [43] and Sparks et. al. [44] showed that shape features of glands on prostate pathology images could predict the Gleason score of the disease. QH features may therefore provide a more informative set of measurements for correlating with *in vivo* imaging features, compared to gross measurements of tissue component areas. For instance, since radiomic features such as Sobel, Kirsch, Gabor, and gradient filters can capture edge orientation information within the image, and Haralick texture features can capture image texture and heterogeneity, this then raises the question of whether these directional and Haralick features that were able to distinguish low- from high-Gleason score PCa are actually capturing fundamental disorder in gland lumen orientation architecture. If a QH feature is determined to be predictive of Gleason score, and it is correlated with a radiomic feature that is also predictive of Gleason score, then it is likely that the QH feature contributes, at least in part, to the discriminability of that radiomic feature. This careful quantitative correlative analysis of radiomic features with QH features extracted from corresponding digitized surgical tissue specimens has not, to the best of our knowledge, been previously done, and could therefore help unearth the cellular basis for why specific radiomic features work.

In this study, we attempt to identify which specific radiomic features on MRI are correlated with specific QH features extracted from hematoxylin and eosin (H&E)- stained *ex vivo* surgical specimens which are in turn predictive of the Gleason grade of PCa. Our study is comprised of digitized images of *ex vivo* surgical prostatectomy specimens and the corresponding pre-operative MRI from prostate cancer patients. We utilize deformable registration to map regions annotated for cancer on digitized *ex vivo* H&E-stained histopathology sections of the surgical specimens onto corresponding preoperative *in vivo* T2w MRI slices. Then, correlative analysis of the radiomic and QH features identified to be most predictive of Gleason score of PCa was performed via three main experiments. First, the top radiomic features most discriminating of low and high Gleason scores are identified on a per-region basis using feature selection over 1000 repetitions of cross-validation. Second, QH features found to be most highly correlated with each of the top discriminating radiomic features identified from Experiment 1 are determined. Multiple hypothesis correction is employed to determine which correlations are statistically significant. Finally the third experiment involves identifying the subset of the most predictive QH features out of those found to be correlated with predictive radiomic features in Experiment 2. The results of each experiment are then validated on an independent dataset from a different institution.

The remainder of the paper is organized as follows. The next section describes the details of patient selection, data preprocessing, and feature extraction. Then, the experimental design is described. Finally, we present and discuss the findings of these experiments in the context of previous work, and end with concluding remarks.

## Methods

### Ethics statement

Data analysis was waived review and consent by the IRB board, as all data was being analyzed retrospectively, after de- identification. All experimental protocols were approved under the IRB protocol # 02-13-42C with the University Hospitals of Cleveland Institutional Review Board, and all experiments were carried out in accordance with approved guidelines. Under this IRB, we were allowed to obtain de-identified images from St Vincent’s Hospital and University of Pennsylvania, and material transfer agreements were signed and agreed upon between Case Western Reserve University and University of Pennsylvania and St. Vincent’s Hospital.

### Data collection and processing

Subjects who underwent 1.5 or 3 Tesla (T) T2w MRI prior to radical prostatectomy at two different institutions were eligible for this study: (1) 54 patients from University of Pennsylvania (UPenn) imaged between 2009 and 2011, and (2) 17 patients from St. Vincent’s Hospital (SV) imaged between 2012 and 2014. Due to funding constraints, 23 out of the 54 patients from UPenn were selected to include a spectrum of cases with different Gleason scores, and all 17 patients from SV were selected for digitization. 4 out of these 17 patients from SV had tumor regions that were too small to be accurately co-registered with MRI, thus were excluded from our study. Dataset 1 (*D*_1_) from UPenn served as our training cohort, and dataset 2 (*D*_2_) from SV served as our independent validation cohort. Details regarding MR scanning and acquisition are summarized in Table 1.

**Table 1:** MR parameters.

Cohort	N	Sequence	Scanner	Echo Time (ms)	Repetition Time (ms)
*D*_1_ (Training)	23	T2w MRI	3T Verio, Siemens	107-127	3690-7090
*D*_2_ (Validation)	11	T2w MRI	3T, Philips Medical Systems	67-100	2525-3567
	2	T2w MRI	1.5T Siemens	119	3760

Each surgically resected prostate gland was fixed in formalin, embedded in paraffin, and sectioned axially in a plane perpendicular to the urethral axis from apex to base in 3-4 mm sections. Each slice was then sectioned into four quadrants, stained with hematoxylin & eosin (H&E), and digitized via an Aperio whole slide scanner at 20X magnification and 0.5 um/pixel resolution. Quadrants were then digitally reconstructed into pseudo whole-mount histology sections (PWMHSs) using previously presented tools. [45–47] A pathologist (R.E.) and radiologist (J.G.) then worked together to identify corresponding T2w MRI and PWMHS slices from the prostate mid-gland alone to help ensure close correspondence. These slices were then co-registered using thin plate splines (TPS), a landmark based deformable registration method. [48] Manually selected landmarks were used to align prostate boundaries and visible internal structures (such as urethra) between the moving histology image and the target T2w MRI image.

Tumor regions were annotated by a pathologist (N.S., 10 years of experience; R.E., 5 years of experience) on digitized H&E stained slides using ImageScope (Aperio) software. A tumor region was defined as any contiguous spatial region containing a distinct tumor, and the manually annotated outline of the tumor region was drawn to encompass the entirety of each tumor region. Therefore, it was possible to identify multiple tumor regions per slide. However, tumor regions smaller than 18*mm*^2^ were excluded from our study, as these regions comprise a small number of pixels on the MR image, which may make the co-registration of these regions less robust and reliable than for larger regions. Pathologists determined Gleason scores for each tumor region (M.F for St Vincents; R.E. for UPenn). Based on these Gleason scores, regions were categorized as either low- (score of 3+3), intermediate- (score of 3+4 or 4+3), or high- (score of 4+4 or higher) risk. Based on this categorization, *D*_1_ consisted of 21 low-, 30 intermediate-, and 14 high-risk prostate cancer regions from 23 patients, while *D*_2_ consisted of 26 low-, 8 intermediate-, and 6 high-risk regions from 13 patients.

### Quantitative histomorphometric feature extraction

Our choice of QH features was based on previous work showing the ability of these features to identify malignancy, discriminate low and high Gleason grades of cancer, and predict biochemical recurrence following surgery. [37–41] The features involved capturing different tumor attributes from tissue specimens such as gland lumen architecture, shape, and angularity, relative areas of different tissue components (lumen, nuclei, epithelium, stroma), and texture.

Accurate segmentation of tissue components is critical to QH feature extraction. [49–56] As a pre-processing step, four different tissue components were segmented from digitized tissue images as follows. Gland lumen were segmented using an automatic region-growing algorithm [39] at 1.25x equivalent microscope zoom. Nuclei vs. cytoplasm, and epithelium vs. stroma were segmented using convolutional neural networks trained at 20x microscope zoom using the approach described in Janowczyk et. al. [57] Epithelium segmentations were combined with nuclei and cytoplasm segmentations to obtain epithelial nuclei and epithelial cytoplasm segmentations.

**Fig 1:**
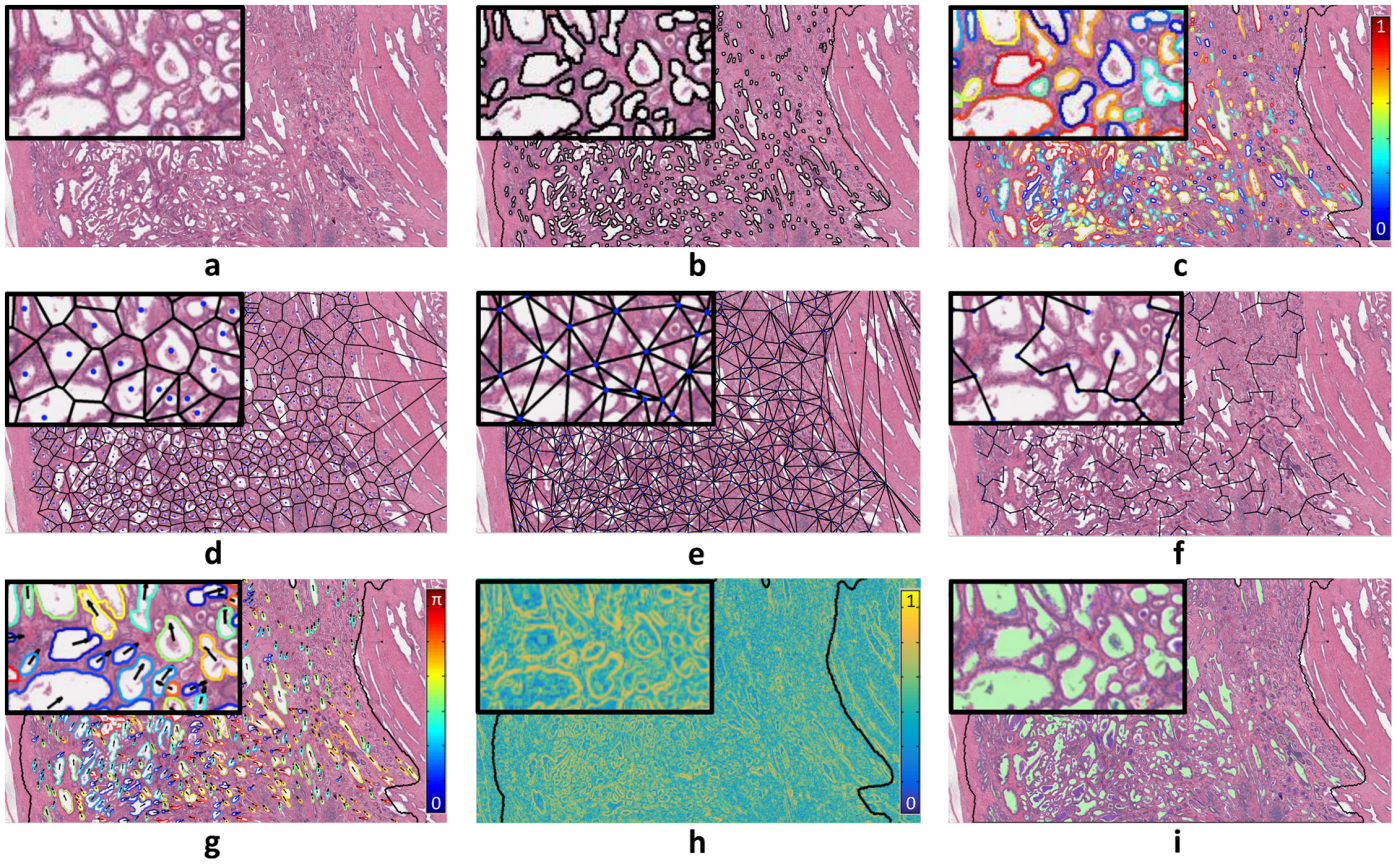
(a) An H&E-stained histology tumor patch. (b) Gland lumen are segmented using an automatic region-growing algorithm within the tumor region. [39] (c) Segmentation boundaries enclosing the gland lumen provide measures of shape, such as Fourier descriptors, which are decompositions that capture various levels of shape complexity, as illustrated here with Fourier descriptor 9 (blue = low values, red = high values). Gland lumen architectural features are extracted from (d) voronoi diagram, (e) delaunay triangulation, and (f) minimum spanning tree. (g) Identification of directional tensors for each segmented gland lumen allows extraction of features such as co-occuring gland angularity [42] disorder (arrows and colors depict tensor orientations from 0 to *π*). (h) Haralick texture features [22] are computed within local rectangular windows within the tumor region, resulting in a texture value for each pixel, with Haralick correlation shown here (blue = low values, yellow = high values). (i) Tissue component densities are computed from gland lumen (green), epithelium (blue), nuclei (yellow), and stroma (uncolored).

Using these tissue component segmentations, a total of 828 QH features were extracted as summarized in Table 2 and illustrated in Fig 1:

- Tissue component density (TCD): For each segmented tissue component, we computed the area of each tissue component divided by the area of the parent tumor region as well as relative ratios of TCD between epiethelium, stroma, and lumen.
- The classes of gland lumen features described in the bullets below were summarized for each tumor region using 15 different descriptive statistics: mean, standard deviation, range, minimum, maximum, mode, median, variance, kurtosis, harmonic mean, skewness, mean, absolute deviation, interquartile range, disorder, min/max.
- Gland lumen shape: a total of 25 statistical measures of shape (e.g., smoothness, fourier descriptors, long/short distance ratio were computed for each gland lumen segmentation perimeter, resulting in a total of 375 unique shape features. [58]
- These features attempt to capture the architectural arrangement of gland lumen using statistical measures of graph characteristics extracted from voronoi diagrams, Delaunay triangulations, and minimum-spanning-tree graphs. [59, 60] The vertices of the graphs are defined as segmented gland lumen centroids. A voronoi diagram is the spanning graph comprised of polygons surrounding all vertices, where each polygon’s edge is equidistant from the nearest two vertices. A Delaunay triangulation is the spanning graph connecting adjacent vertices to form triangles, where no points are within the circumcircle of any triangle. A minimum-spanning-tree is the spanning graph connecting vertices with the smallest possible total edge lengths.
- Nearest-neighbor (NN) architectural: These features quantify the number of gland lumen within specified radii, as well as the minimum radii needed to enclose different numbers of gland lumen.
- Co-occuring gland angularity (CGA) [42] features: The dominant orientation of each gland lumen is identified, then co-occurrence features capturing the statistical relationships between neighboring lumens are computed.
- Haralick texture features attempt to capture textural appearance based on statistical relationships between gray levels of neighboring pixels. [22]
- Cell cluster graph (CCG) [61] features: These features compute graph-based architectural statistics from localized sub-graphs within tumor regions.

**Table 2:** List of extracted QH features.

Modality	Type	Features	No. of Features
H&E stained histology	Tissue Component Density (TCD)	Lumen, epithelium, stroma, nuclei, epithelial nuclei, and epithelial cytoplasm fractions. Epithelium/stroma, epithelium/lumen, lumen/stroma ratios.	9
	Shape	Area ratio, distance ratio, standard deviation of distance, variance of distance, long/short distance, perimeter ratio, smoothness, invariant moments 1-7, fractal dimension, fourier descriptors 1-10. [58]	375
	Architectural: Voronoi diagram	Polygon area, perimeter length, and chord length. [59, 60]	45
	Architectural: Delaunay triangulation	Triangle area and side length. [59, 60]	30
	Architectural: Minimum spanning tree (MST)	Edge length. [59, 60]	15
	Architectural: nearest neighbors (NN)	(a) Distance to 3, 5 and 7 NN and (b) number of NN in 10, 20, 30, 40, 50 pixel radius (PR).	120
	Architectural	Area, number, and density of polygons.	3
	Texture	Contrast energy, contrast inverse moment, contrast average, contrast variance, contrast entropy, average, variance, entropy, energy, correlation, information measure 1, information measure 2. [22]	195
	Co-occuring Gland Angularity (CGA) [42]	Contrast energy, contrast inverse moment, contrast average, contrast variance, contrast entropy, average, variance, entropy, energy, correlation, information measure 1, information measure 2.	195
	Cell Cluster Graph (CCG) [61]	Number of Nodes, Number of Edges, Average Degree, Average Eccentricity, Diameter, Radius, Average Eccentricity 90%, Diameter 90%, Radius 90% Average Path Length, Clustering Coefficient C, Clustering Coefficient D Clustering Coefficient E, Number of connected components, giant connected component ratio average connected component size, number isolated nodes, percentage isolated nodes number end nodes, percentage end nodes, number central nodes, percentage central nodes mean edge length, standard deviation edge length, skewness edge length, kurtosis edge length.	37

### Radiomic feature extraction

Our choice of radiomic features was based on previous work demonstrating their promise for detecting and discriminating between less and more aggressive PCa. [4–7, 25–28] These features are based on quantifying the following different image characteristics: signal intensity, texture, and edge orientation.

**Fig 2:**
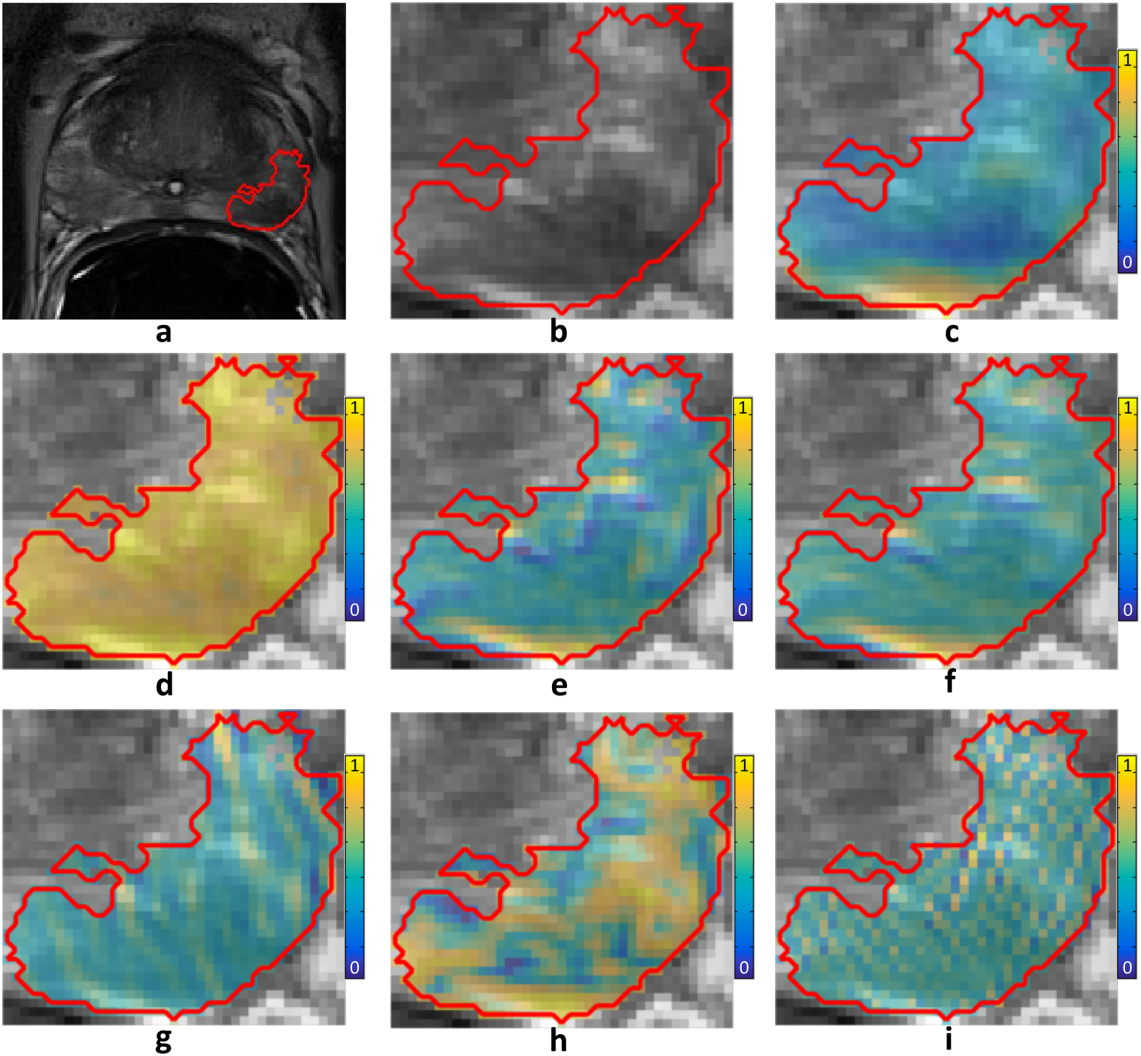
(a) A T2w MRI of the prostate (tumor outlined in red). (b) Zoomed-in view of tumor. Sample radiomics feature maps from each class of radiomic features are plotted (blue = low, yellow = high). (c) Standard deviation within 9x9 windows. (d) Entropy within 5x5 windows. (e) Sobel yx filter. (f) Kirsch 1 filter. (g) Gabor cosine filter at an angle of $\frac{\pi }{8}$ and wavelength 4. (h) Haralick correlation within 5x5 windows. (i) Laws 5x5 spot-ripple filter.

**Fig 3:**
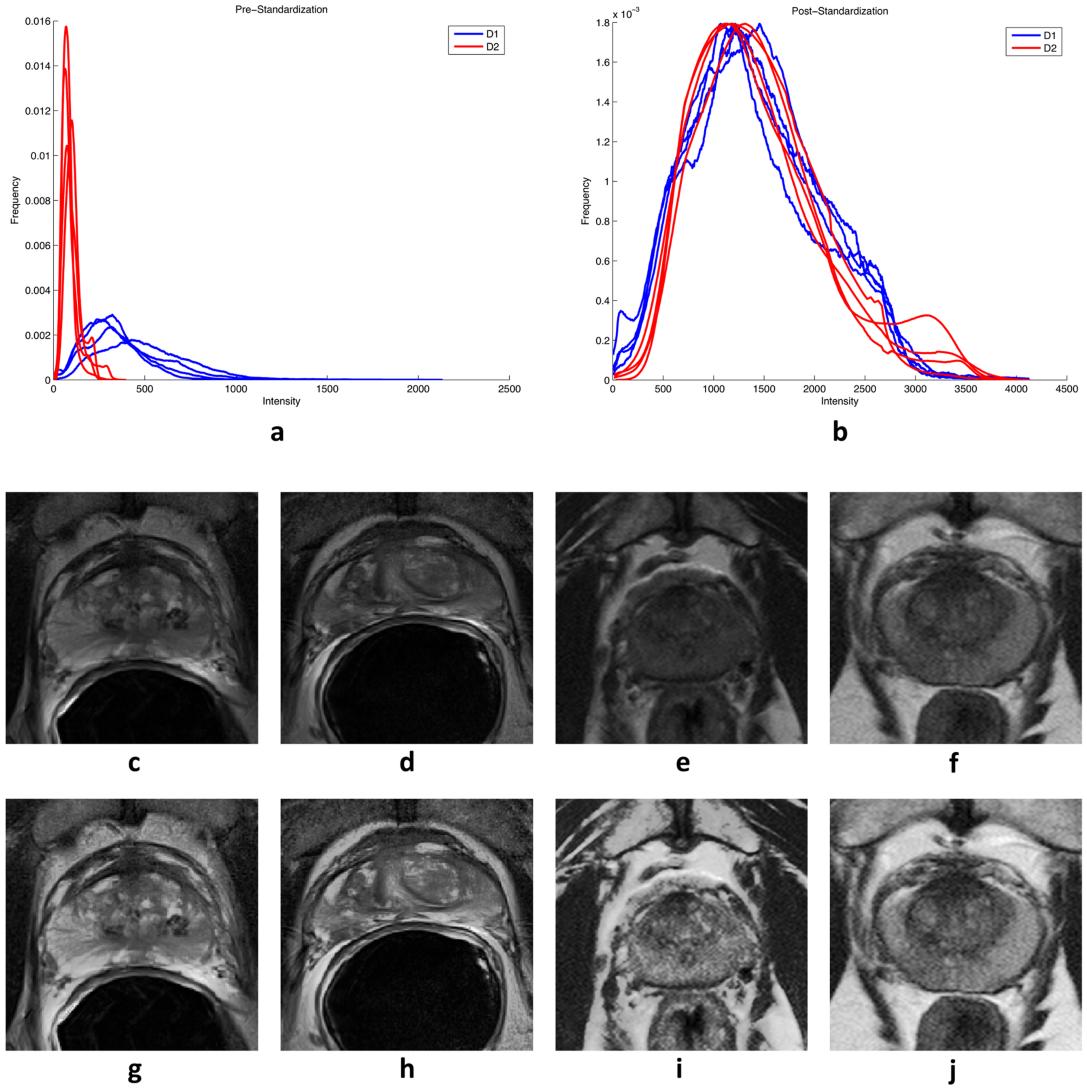
(a) T2w MRI intensity distributions prior to standardization for four patients from *D*_1_ (blue) vs. four patients *D*_2_ (red), demonstrating poor inter- and intra-institutional alignment between intensity distributions of different patients. (b) After standardization to a template constructed from *D*_1_, the intensity distributions demonstrate better alignment. (c-f) Sample T2w MRI images pre-standardization and (g-j) post-standardization for (c,d,g,h) two patients from *D*_1_ and (e,f,i,j) two patients from *D*_2_.

Prior to feature extraction, T2w MRI signal intensity was corrected for intensity non-standardness, which is a well-known issue in MRI that causes voxel intensity values to not have a fixed tissue-specific meaning. [62] To correct for intensity non-standardness, T2w MRI images from the training and validation cohorts were standardized to a template distribution, defined as the per-patient median of intra-prostatic pixel intensities from the training cohort, using the algorithm presented by Nyul et. al., [63] as illustrated in Fig 2. Then, radiomic features were extracted using a large set of co-occurrence methods and convolutional filter kernels, including Gabor, Haralick, and Laws features, as illustrated in Fig 3. A total of 157 2D radiomic feature responses were computed, and then summarized for each tumor region using 13 different descriptive statistics (mean, standard deviation, range, minimum, maximum, mode, median, variance, kurtosis, harmonic mean, skewness, mean, absolute deviation, interquartile range), resulting in a total of 2001 radiomic features, as summarized in Table 3.

**Table 3:** List of extracted radiomic features.

Modality	Type	Features	No. of Features
T2w MRI	Signal intensity	Voxel intensity.	13
	Descriptive statistics of signal intensity	Mean, median, standard deviation, range, within windows of size: 3x3, 5x5, 7x7, 9x9.	208
	Entropy	Entropy within window sizes of 3x3, 5x5, 7x7, 9x9.	52
	Gradient features and gradient-like kernel operations	Gradient (x, y, magnitude), dx, dy, ddiag, Sobel (x, y, xy, yx), Kirsch 1-3.	169
	Gabor	Sine, cosine, and magnitude abor filter responses for *θ* ∈ [0: *π*/8: *π*] × λ ∈ [2, 3, 4].	936
	Haralick texture [22]	Contrast energy, contrast inverse moment, contrast average, contrast variance, contrast entropy, average, variance, entropy, energy, correlation, information measure 1, information measure 2 within windows of size 3x3 and 5x5.	338
	Laws texture energy [64]	Laws texture energy 5x5 kernels (25 total).	325

## Experimental design

We performed the following three experiments, described below:

**Experiment 1: Identifying radiomic features to discriminate low and high Gleason score tumors on T2w MRI:** The set of radiomic features most discriminable of Gleason score was identified by performing minimum redundancy maximum relevance (mRMR) feature selection [65] on *D*_1_. To reduce the likelihood of over-fitting the predictive model to the training data, the top seven (roughly one tenth of the number of samples) most frequently selected features from one thousand repetitions of three-fold cross-validation (CV) were identified based on voting across all repetitions of CV. In each repetition of CV, classification performance of the selected features was quantified to obtain an initial estimate of the predictive potential using a random forest (RF) classifier, which has been shown to be most prognostic and stable classifiers for radiomic features. [66]

The final set of seven features identified was then used to retrain a RF classifier on *D*_1_ (no CV). The classifier’s generalizability was then evaluated by testing it on *D*_2_.

**Experiment 2: Identifying QH features correlated with the most predictive radiomic features identified in Experiment 1:** To identify the set of QH features that might form a morphologic basis for radiomic features, the pairwise correlation between each of the top radiomic features selected in Experiment 1 and each of the 828 QH features was computed on *D*_1_, resulting in a total of 5796 pairs of features being tested for correlation.

The pairs of features identified as being correlated were then re-tested on *D*_2_ to determine if the correlations still held on an independent cohort. This yielded a set of QH and radiomic features that appeared to be strongly correlated with each other across datasets from two different sites.

**Experiment 3: Identifying the QH features most discriminating of low and high Gleason score disease from among the set identified in Experiment 2:** The subset of QH features identified in Experiment 2 most discriminating of low and high Gleason score were identified on *D*_1_, via mRMR. As in Experiment 1, these features were identified based on voting across all 1000 repetitions of CV. In each repetition of CV, classification performance of the top selected features was quantified to obtain an initial estimate of the predictive potential using a random forest (RF) classifier.

The final set of seven features identified was then used to retrain a RF classifier on *D*_1_ (no CV). The classifier’s generalizability was then evaluated by testing it on *D*_2_.

### Statistical evaluation

**Selection frequency to identify top QH or radiomics features:** Selection frequency for each feature was computed as the total number of folds in which the feature was selected among the top ranked features for that training fold, divided by the total number of training folds. The top ranked radiomic and QH features were selected by choosing those with the highest selection frequencies over all repetitions of CV.

**AUC to evaluated classifier performance:** Receiver operating characteristic (ROC) curves were used to evaluate classification performance. The overall ROC and upper- and lower-bound curves were computed as the mean of curves over all repetitions of CV. Upper and lower bounds were computed using threshold averaging within each repetition of CV.

**Multiple hypothesis correction to evaluate correlations:** A correlation coefficient and associated p-value were computed for each pair of features using Spearman’s rank correlation test, which is robust to outliers and quantifies monotonicity. [67] Statistically significantly correlated features were identified using False Discovery Rate (FDR) for multiple hypothesis testing. [68] On the training set and validation set, statistically significant correlations were identified as those with FDR <= 0.05 and FDR <= 0.2, respectively.

## Results

**Fig 4:**
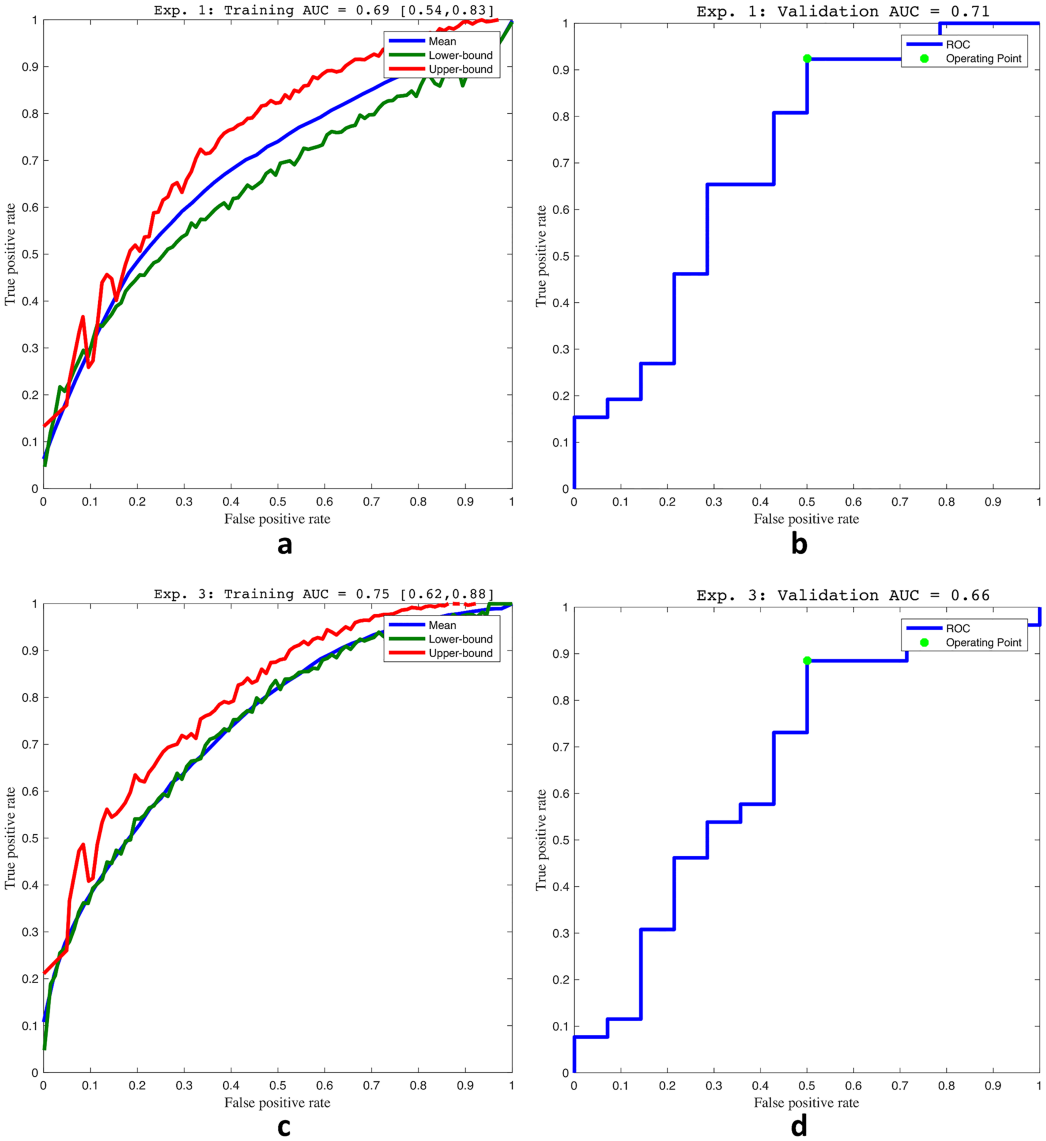
Receiver operating characteristic (ROC) curves depicting random forest classification performance for discriminating low from intermediate/high Gleason score. (a) training on *D*_1_ and (b) validation on *D*_2_ of radiomic features for Experiment 1. (c) training on *D*_1_ and (d) validation on *D*_2_ of QH features for Experiment 3.

**Experiment 1**: The top selected radiomic features and their selection frequencies are listed in Table 4, and ROC curves of their discriminability on the training and validation cohorts are shown in Fig 4a and 4b. This feature subset comprises 3 Gabor, 2 Laws, 1 Haralick texture, and 1 descriptive statistic feature, which were able to distinguish low from intermediate and high Gleason score prostate cancer tumors with an AUC of 0.69 on the training cohort and 0.71 on the validation cohort, as shown in Fig 4.

**Table 4:** Selected radiomic features. *Features significantly correlated on the independent validation dataset.

Radiomic Feature	Selection Frequency (%)
Gabor:sin:theta = 1.1781:lambda = 2:Mean	40.1
Laws15:Mean	38.5
Haralick:correlation:ws5:Maximum	37.3
Laws14:Kurtosis*	21.0
Median:WindowSize7:Maximum	20.3
Gabor:cos:theta = 0.3927:lambda = 4:Variance*	19.7
Gabor:sin:theta = 0:lambda = 2:Median	19.2

**Fig 5:**
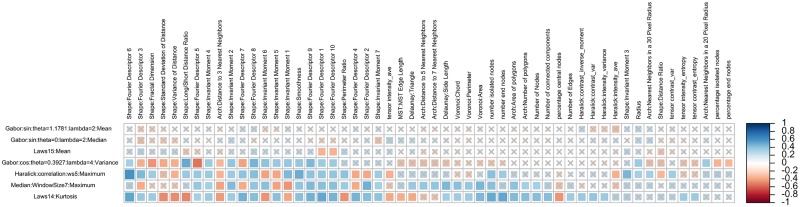
The maximum Spearman’s rank correlation coefficients for each QH feature group (columns) and each discriminating radiomic feature (rows) selected in Experiment 1 are plotted (red = negative correlation, blue = positive correlation), with gray X’s denoting lack of statistical significance.

**Fig 6:**
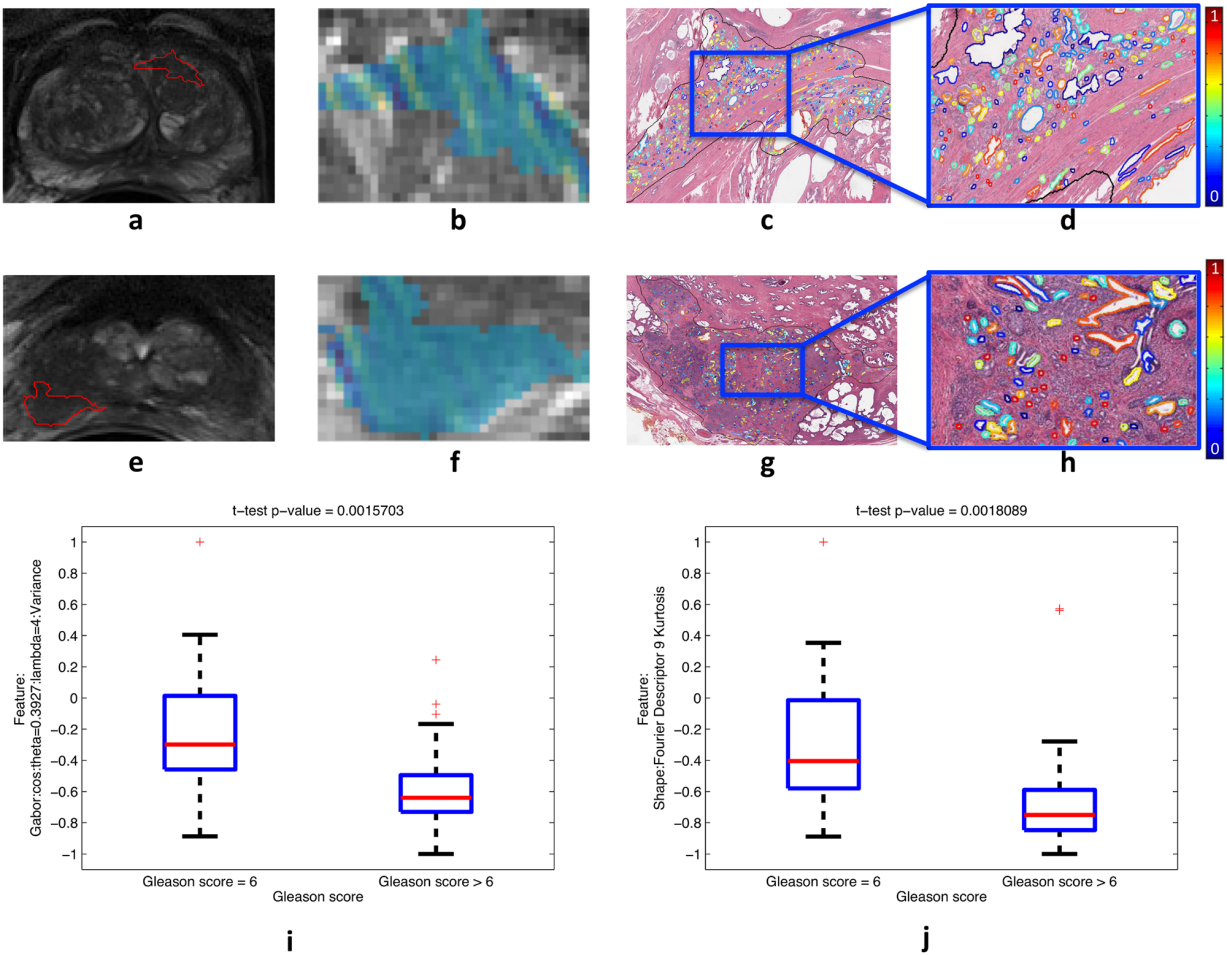
Differential expression for a pair of strongly correlated and discriminating radiomic and QH features between tumors with Gleason score 6 (a-d) and Gleason score 9 (e-h) from *D*_1_. The radiomic feature values are plotted within the tumor (blue = low values, yellow = high values) shown within the whole prostate (a,e) as well as zoomed-in (b,f). The corresponding QH feature values are plotted within the tumor region on the segmented gland lumen boundaries (blue = low values, red = high values), shown within the whole tumor (c,g) as well as zoomed-in (d,h). Boxplots summarizing the differential expression of these features over all tumors from *D*_1_ (N = 65) are plotted (i, r). Note the low number of colors in the tumor with high (f) relative to low (b) Gleason score, indicating lower variance of the depicted Gabor feature within the high Gleason score tumor. Additionally, note the greater number of colors in the tumor with high (h) relative to low (d) Gleason score, indicating lower kurtosis of the depicted shape feature within the high Gleason score tumor.

**Experiment 2**: Of the 5796 pairs of QH and discriminating radiomic features, 401 pairs consisting of 234 unique QH features were statistically significantly correlated on the training cohort (FDR <= 0.05). These correlations are visualized in Fig 5, which illustrates the maximum correlation coefficient for each QH feature group. These 401 correlations were then tested on the independent validation cohort, resulting in 56 out of 401 statistically significant (FDR <= 0.2) correlations. Three of the 56 pairs of features contained radiomic and QH features that were both selected among the top discriminating features in Experiments 1 and 3, as marked with asterisks in Tables 4 and 5. The differential expression between low and intermediate/high Gleason score tumors for one of these pairs of features is illustrated in Fig 6.

**Table 5:** Selected QH features. *Features significantly correlated on the independent validation dataset.

QH Feature	Selection Frequency (%)
Shape: Fourier Descriptor 9 Median	59.9
Shape: Fourier Descriptor 3 Minimum	59.7
Shape: Fourier Descriptor 5 Disorder	43.3
Shape: Fractal Dimension Mean	40.8
Shape: Fourier Descriptor 9 Kurtosis*	36.1
Shape: Invariant Moment 6 Interquartile Range*	34.7
Shape: Fractal Dimension Maximum*	33.6

**Experiment 3**: The top 7 QH features selected from the set of 401 unique correlated QH features identified in Experiment 2 are listed in Table 5. This feature subset comprises 7 shape features, which were able to distinguish low from intermediate and high Gleason score prostate cancer tumors with an AUC of 0.75 on the training cohort and 0.66 on the validation cohort, as shown in Fig 4. Since these features are both discriminating of low and high Gleason score and correlated with discriminating radiomic features, they may therefore provide the morphologic basis and justification to help explain the discriminability of the radiomic features identified in Experiment 1.

## Discussion

Radiomics may be predictive of Gleason score; [5] where the latter provides the current clinical gold standard for assessing the risk of PCa metastasis [69]. Thus apart from confirming presence or absence of disease *in vivo*, radiomics could also enable non-invasive risk stratification and early identification of patients who might be better candidates for active surveillance compared to definitive therapy.

There have been some recent works looking at radiomic features from prostate MRI which are predictive of Gleason score *in vivo*. [5, 6, 28, 70] Previously, Haralick texture features as well as edge orientation features such as Sobel, Kirsch, and gradient features have been found to be discriminating of low and high Gleason score. [5, 6, 28, 70] However none of these studies have explicitly sought to identify the cellular or morphologic basis of why these features have been found to be predictive of Gleason score. Since radiographic appearance of disease is a reflection of the morphometric appearance of disease at the tissue level, it stands to reason that radiomic features identified as predictive on the imaging length scale must have a morphologic basis at the cellular length scale.

In this study, we sought to identify the QH features that reflect the morphologic basis of radiomic features predictive of different Gleason scores of prostate cancer. *Ex vivo* radical prostatectomy histopathology images were carefully co-registered with pre-operative MRI to map the gold-standard disease annotations onto MRI. Comprehensive sets of radiomic and quantitative histomorphometric features were spatially co-localized to characterize each tumor region. This framework enabled us to robustly identify correlated sets of these features, which were also predictive of cancer risk.

Our framework attempted to exploit the fact that if a QH feature is discriminating of Gleason score, and it is correlated with a radiomic feature that is also predictive of Gleason score, then it is likely that the QH feature provides, at least in part, the morphologic basis for the discriminability of said radiomic feature. To validate our findings, the features identified in each of the three experiments were independently evaluated on a test dataset from a different institution.

This study identified a set of T2w MRI radiomic features as well as a set of QH features correlated with these T2w derived radiomic features, which were both identified to be predictive of Gleason score. While it is difficult to demonstrate perfectly that there is a causal relationship between these features, we designed our experiments in such a way as to expect some form of causal relationship between these features to exist. For instance, the relationships between some of the features identified in this study appear to have plausible biophysical explanations. It is well-documented that as Gleason grade increases, the gland lumen shrinks and the orientations become more disorderly. [69] The Gleason grading system provides the current clinical gold standard prognostication of prostate cancer. The system enables stratification of patients into different risk groups based solely on the architectural patterns of gland units present in their pathology specimens. In normal prostate tissue specimens, this architecture is characterized by circular or branching glands separated by fibromuscular stroma. These normal glands consist of a lumen space, which facilitates the flow of prostatic secretions, surrounded by a ring of epithelial cells, resulting in a well-differentiated appearance. In cancerous tissue, this gland architecture is disrupted. In lower Gleason grades (3 or lower), glands begin invading the surrounding stroma. In more aggressive, higher grade disease (grades 4 and 5), this architecture breaks down further, resulting in a poorly differentiated appearance in which the gland lumen appear more chaotic and scattered, and may no longer be fully surrounded by epithelial cells. Thus, gland lumen shape features may provide an intuitive choice for capturing the architectural breakdown of glands in the cancerous prostate. [69, 71] The set of QH features discriminating of low and high Gleason score that were identified in Experiment 3 are primarily reflective of grade-related changes in gland lumen shape, as all 7 selected QH features are gland shape features.

In our experiments we identified a Gabor texture filter to be most predictive of Gleason score as well as being highly correlated with gland lumen shape features. Gabor filters represent a convolution of an exponential and a sinusoidal function, which enables capturing of MR image gradients across different scales and orientations. On T2-weighted magnetic resonance imaging (MRI), prostate cancer is characterized by a darker (lower intensity), mass-like appearance. Recent work has shown strong associations between cellular level tumor features, such as lumen-space area and nuclear area, and radiographic measurements of T2 intensity. [33–35] Although gland lumen are typically substantially smaller than the size of a voxel on MRI, we hypothesize that the differential Gabor filter response for low and high Gleason score tumors may be due to the cumulative contributions of gland lumen shape differences between these 2 types of tumors. In other words, the more prominent gland lumen which are characteristic of lower Gleason score tumors might be the cause of a strong Gabor response along specific orientations. By contrast, the differentially sized gland lumen associated with higher grade tumors might be the reason for lack of a coherent Gabor signal along any orientation. We must note that both the correlation between Gabor responses and gland shape as well their predictive ability have been tested and evaluated on independent testing and validation sets. This allows us reasonable confidence in suggesting that the progressive breakdown of gland architecture and morphology in higher grade tumors may be responsible for the differential Gabor signal on MRI for low and high Gleason score tumors.

Our study, however, did have its limitations. First, the datasets we used were relatively small. This had to do with constraints due to both time and budget since each of the surgical specimens had to be meticulously sectioned, stained, digitized and annotated for this study. Second, there is no practical method for assessing registration accuracy at a per-voxel level, although attempts were made to minimize errors in the *x* − *y* plane by selecting anatomical landmarks visible on both histology and MRI. Additionally, identifying slice correspondences to ensure accurate mapping in the *z* plane is a highly challenging task. Third, while diffusion-weighted (DW) and dynamic contrast-enhanced (DCE) have been shown to be highly promising for prediction of Gleason score, it was not included in our study because DW and DCE images were not available for both our training and validation cohorts. It has been previously found that architectural features of blood microvessels are correlated with kinetic DCE features, as well as discriminative of low vs. high Gleason scores. This in turn suggests that blood vessel irregularity may provide the morphologic basis for radiomic DCE features in prostate cancer. [32]

In order to develop predictive models that can be successfully applied to unseen data from different sites and scanners, it is essential to use reliable, reproducible pathomic features. In this study, we have carefully selected a pool of pathomic features that have been used to successfully train predictive models for a variety of disease contexts. Although we have not quantitatively evaluated the reliability and stability of the features used in our study for the task of Gleason grading, there are several additional sources of indirect evidence that point to the reliability of the features we used: (a) these features have been used successfully in related domains of cancer prognosis prediction, (b) these features have plausible biological relevance, and (c) these features have been used in collaboration with pathologists [42, 59–61, 72]. Furthermore, this issue should also be considered in the context of the difficulty and complexity of Gleason grading, which is known to have high inter- and intra- observer variability among experienced pathologists [73]. As computational methods involving pathomic features move closer to widespread clinical use [36], it will be essential to gain a more comprehensive understanding of how these features are affected by differences in acquisition and scanning between different sites. In a recent study, Leo et. al. [74] measured the extent to which pathomic features are affected by differences in acquisition between clinical sites and tissue slide scanners, for similar prostate cancer patient populations (controlled for Gleason score and patient outcome). They found that pathomic feature values differ significantly between sites, and that these differences affect the ability of these features to generalize across datasets from different clinical sites for the task of automated tumor detection. Some families of features were found to be more stable, and to generalize better than other families of features. In particular, Gland lumen shape features were found to be the most stable, and Haralick texture features were found to be the least stable. We find it reassuring that all of the top 7 most QH features most predictive of Gleason grade identified in our study are Gland lumen shape features.

Future directions envisioned for this work include evaluating the associations identified in this study on larger data cohorts from multiple institutions, as well as evaluating their ability to predict disease outcome as opposed to Gleason grade alone. In addition, we would like to explore associations between radiomic features from other multiparametric MRI protocols such as diffusion-weighted and dynamic contrast-enhanced MRI which might in turn enable better predictions of prostate cancer risk and aggressiveness.

## Concluding remarks

In this work we identified promising associations between *in vivo* T2w MRI radiomic features capable of predicting prostate cancer risk and quantitative histomorphometric features, thus providing potential explanations for the morphologic basis of some of these radiomic features. Specifically we identified that Gabor filters may be highly predictive of Gleason score on MRI, and these filters may be responding to grade-related differences in gland lumen shape within tumor regions.
